# PCSK9 inhibitors ameliorate arterial stiffness in ACS patients: evidences from Mendelian randomization, a retrospective study and basic experiments

**DOI:** 10.3389/fmed.2024.1408760

**Published:** 2024-05-27

**Authors:** Linghao Xu, Liang Wang, Yuanqi Wang, Yiqiong Wang, Yuanzhen Jiang, Peizhao Du, Jing Cheng, Chunsheng Zhang, Ruijie Wang, Tiantian Jiao, Lijian Xing, Jiangping Ma, Jiming Li

**Affiliations:** ^1^Department of Cardiology, Shanghai East Hospital, School of Medicine, Tongji University, Shanghai, China; ^2^Shanghai University of Medicine and Health Sciences Affiliated Zhoupu Hospital, Shanghai, China; ^3^Department of Cardiology, Baoshan District Hospital of Integrated Traditional Chinese and Western Medicine of Shanghai, Shanghai University of Traditional Chinese Medicine, Shanghai, China; ^4^Department of Cardiology, East Hospital of Clinical Medical College, Nanjing Medical University, Nanjing, China; ^5^Department of Cardiology, Harbin Medical University First Affiliated Hospital, Harbin, China; ^6^School of Medicine, Tongji University, Shanghai, China

**Keywords:** acute coronary syndromes, PCSK9 inhibitors, arterial stiffness, C-reactive protein, Mendelian randomization

## Abstract

**Background:**

Current evidences suggest that Proprotein Convertase Subtilisin/kexin Type 9 inhibitors (PCSK9i) exhibit a protective influence on acute coronary syndrome (ACS). Nevertheless, further investigation is required to comprehend the impact and mechanisms of these pharmaceutical agents on inflammatory factors and arterial stiffness (AS) in patients with ACS. Consequently, the objective of this study is to ascertain the influence of PCSK9i on arterial stiffness in ACS patients and elucidate the underlying mechanisms behind their actions.

**Methods:**

This study employed Mendelian randomization (MR) analysis to examine the association between genetic prediction of PCSK9 inhibition and arterial stiffness. Data of 71 patients with ACS were retrospectively collected, including PCSK9i group (*n* = 36, PCSK9 inhibitors combined with statins) and control group (*n* = 35, statins only). Blood lipid levels, inflammatory markers and pulse wave velocity (PWV) data were collected before treatment and at 1 and 6 months after treatment for analysis. Additionally, cell experiments were conducted to investigate the impact of PCSK9i on osteogenesis of vascular smooth muscle cells (VSMCs), utilizing western blot (WB), enzyme-linked immunosorbent assay (ELISA), and calcification index measurements.

**Results:**

The results of the MR analysis suggest that genetic prediction of PCSK9 inhibition has potential to reduce the PWV. Following treatment of statins combined with PCSK9 inhibitors for 1 and 6 months, the PCSK9i group exhibited significantly lower levels of total cholesterol (TC), low-density lipoprotein cholesterol (LDL-C), C-reactive protein (CRP), interleukin-6 (IL-6), fibrinogen (FIB) and procalcitonin (PCT) compared to the control group (*p* < 0.05). Additionally, PWV in the PCSK9i group demonstrated significant reduction after 6 months of treatment and was found to be associated with the circulating CRP level. In cell experiments, PCSK9i pretreatment ameliorated osteogenesis of VSMCs through reducing the deposition of calcium ions, alkaline phosphatase (ALP) activity, and expression of runt-related transcription factor 2 (RUNX2).

**Conclusion:**

PCSK9i have potential to enhance arterial stiffness in ACS patients. Specifically, at the clinical level, this impact may be attributed to alterations in circulating CRP levels. At the cellular level, it is associated with the signaling pathway linked to RUNX2.

## 1 Introduction

Acute coronary syndromes (ACS) encompass a collection of clinical syndromes characterized by the rupture or invasion of coronary atherosclerotic plaques, resulting in the formation of complete or incomplete occlusive thrombi (1). ACS includes acute ST-segment elevation myocardial infarction (STEMI), acute non-ST-segment elevation myocardial infarction (NSTEMI), and unstable angina (UA) (1). Several common contributing factors include the process of aging, smoking, dyslipidemia and glycemia disorders, as well as hypertension (2). In clinical practice, various pharmacological treatments including antiplatelet therapy, lipid modulation, blood pressure control, and glucose therapy, alongside revascularization therapies such as percutaneous coronary intervention (PCI) and heart bypass surgery are commonly employed (3), within 10 years, more than 20% of patients with ACS are at more than a 30% risk of recurrence which can lead to myocardial infarction, stroke, and other vascular-related fatal events (4, 5).

Lipid-lowering agents are presently employed as one of the primary therapeutic intervention in clinical pharmacology for the majority of ACS patients (3, 6). Proprotein Convertase Subtilisin/kexin Type 9 inhibitors (PCSK9i) have primarily been employed for the purpose of reducing levels of low-density lipoprotein cholesterol (LDL-C) in the bloodstream, thereby exerting an anti-atherosclerotic impact (7). The status of PCSK9i which possess robust lipid-lowering properties is progressively growing within the most recent clinical guidelines for ACS (8). Numerous studies have elucidated that PCSK9 inhibitors possess potential broader physiological functions, beyond their established role in lipid level regulation (9).

Arterial stiffness (AS) serves as a significant predictor of cardiovascular disease (CVD) and cardiovascular adverse events. In patients with ACS, elevated AS emerges as a risk factor contributing to unfavorable prognosis. Different from atherosclerosis which is mainly due to vascular endothelial lipid deposition (10), the primary features of AS encompass intravascular collagen deposition, calcium deposition, fibroblast proliferation, and diminished endothelial diastolic function (11). The augmentation of AS may result in a decrease in myocardial afterload and coronary perfusion pressure, consequently precipitating a cascade of severe implications including hypertension, heart failure, and organic heart disease (12). The methods of assessing AS include pulse wave velocity (PWV), ankle brachial index (ABI), ambulatory arterial stiffness index (AASI) and so on, among which the most widely used in clinical practice is PWV (13). Atherosclerosis can be treated with lipid-lowering drugs, but there is currently no drug available for treating AS in clinical settings. Recognizing the presence of AS in patients with ACS and promptly intervening is considered a potential strategy to enhance the prognosis of ACS patients. While the efficacy of PCSK9i in improving AS has been demonstrated in individuals with familial hypercholesterolemia (HF) (14), it remains uncertain whether these inhibitors can exert a similar effect in ACS patients. Given the extensive utilization of PCSK9i in ACS patients, our objective was to investigate the potential of these inhibitors in ameliorating AS in this specific population.

Mendelian randomization (MR) is an analytical approach that employs genetic variations as instrumental variables (IVs) to investigate the potential association between exposures and outcomes (15). Mendelian laws of inheritance randomly divide alleles among offspring, creating equal experimental and control groups. This minimizes confounding factors and makes MR studies a powerful tool for disease and clinical research (16).

Previous studies have mostly focused on clinical trials, but this study represents the first instance of utilizing MR analysis to investigate the correlation between genetic prediction of PCSK9 inhibition and AS. Furthermore, the research not only gathered clinical data from ACS patients treated with PCSK9i and statins but also conducted cell experiments to examine the influence of PCSK9i on the osteogenesis of vascular smooth muscle cells (VSMCs). These approaches offers a more comprehensive insight into the mechanisms of PCSK9i in ameliorating AS.

## 2 Materials and methods

### 2.1 MR design

**Figure 1:**
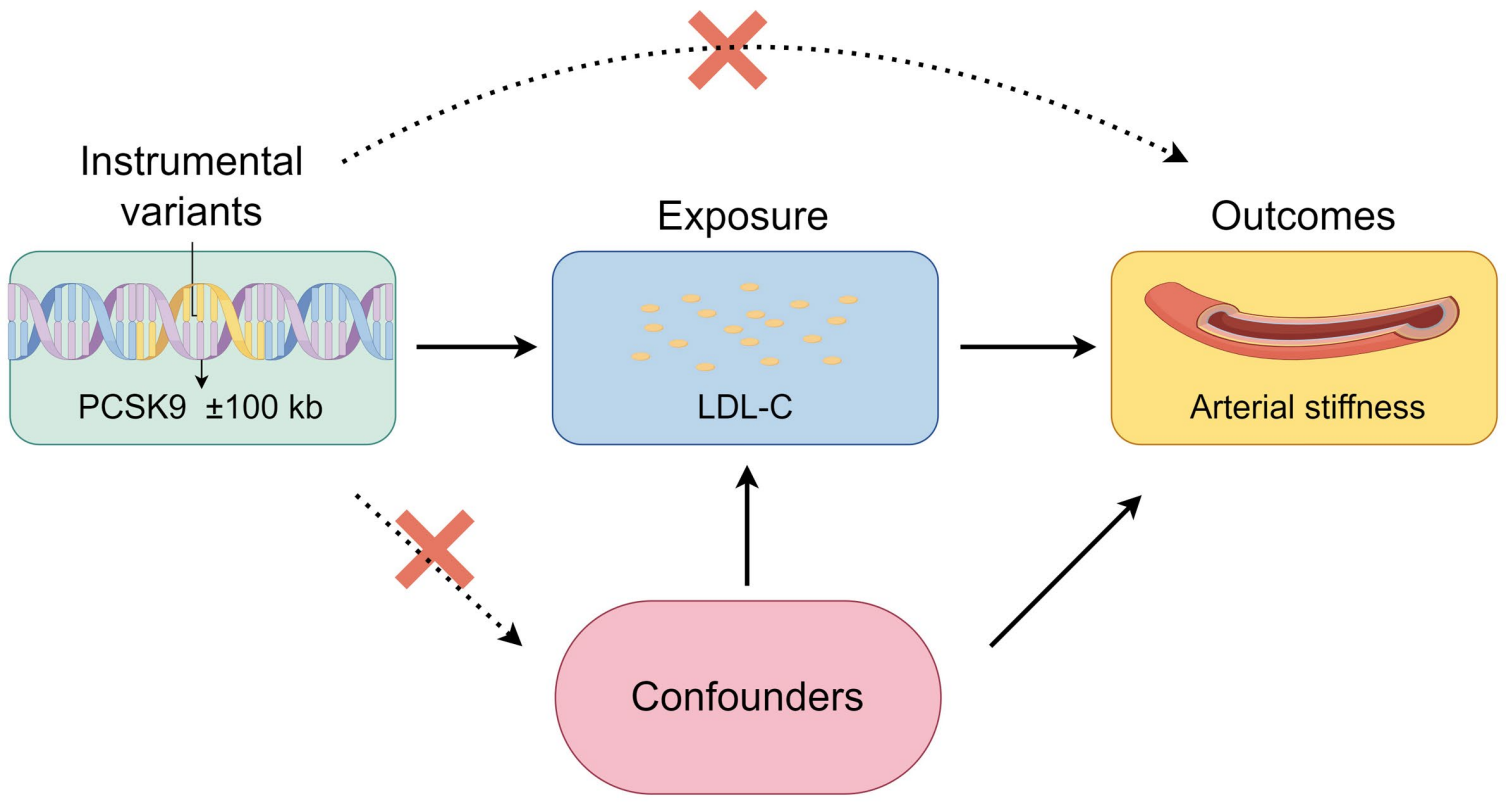
Flowchart of MR design (Draw by figdraw, ID: TRRYUf1311).

Since the clinical effect of PCSK9i on LDL-C reduction is clear, we employed single nucleotide polymorphisms (SNPs) associated with LDL-C as IVs. To establish a causal relationship, it was necessary to satisfy the following criteria: (1) The IVs have no association with confounding factors; (2) The IVs are associated with exposures; (3) The IVs have no direct relationship with outcomes (Figure 1).

### 2.2 Exposure datasets selection and SNPs identification

Genetically predicted inhibition of PCSK9 was used as an exposure. The gene sequence of PCSK9 was searched using the gene search function of the National Center for Biotechnology Information (NCBI) database (17), and the gene region chr1: 55505221–55530525 was selected to identify SNPs that serve as proxies for PCSK9. Utilizing a similar approach for the selection of genetic variants as employed in prior research (18), SNPs were identified within ±100 kb regions of the corresponding gene sequences and were identified in the genome-wide association study (GWAS) LDL-C dataset (ieu-a-300, *n* = 173,082) from Global Lipid Genetics Consortium (GLCC) (19). SNPs that met the genome-wide significance threshold of *p* < 5 × 10–8 and a linkage disequilibrium (LD) threshold of *R*^2^ < 0.2 were utilized as genetic tools. Additionally, the human genotype–phenotype association database PhenoScanner^1^ was employed to identify traits that were directly linked to the SNPs used as IVs. This approach allowed us to exclude potential confounders from our analysis.

### 2.3 Positive control and outcome dataset selection

Based on previously researches (20), we used coronary heart disease (CHD, ieu-a-7) data from the Consortium for the Genetics of Coronary Artery Disease (CARDIoGRAMplusC4D) (21) as a positive control dataset, comprising 60,801 cases and 123,504 controls. Using the pulse wave reflection index (PWRI, ukb-b-11598, *n* = 151,546) from the UK Biobank (UKBB) as the primary outcome, the pulse wave peak to peak time (PWPPT, ukb-b-8778, *n* = 151,466) and pulse wave arterial stiffness index (PWASI, ukb-b-11971, *n* = 151,053) as secondary outcomes. All three parameters serve as indicators of AS, where elevated values correspond to increased levels of AS (22). Datasets were chosen from European populations, although the sources of exposure and outcomes differed. Before the collection of the original database, informed consent was acquired from each participant and approved by the respective local ethics committees of GWAS. All datasets were procured via IEU OpenGWAS (Supplementary Table S1) (23).

### 2.4 MR analysis

Heterogeneity among SNPs was assessed through the application of Cochran’s *Q* test. A *p*-value>0.05 was indicative of the absence of significant heterogeneity. The exposure-related drug-targeting instrument variables were aligned with the outcome dataset and subsequently subjected to analysis employing MR Egger, weighted median, and inverse variance weighted (IVW) methods. MR-PRESSO method was employed to detect potential outliers and address any horizontal polytomous outliers in the analyses. To mitigate the influence of individual SNPs on our findings, we employed the leave-one-out approach, systematically excluding each SNP to ascertain its lack of association with the outcome or presence of pleiotropy. All MR results are expressed as OR or beta, and statistical tests were two-sided. Analyses were completed using R language software (4.2.3), and the TwoSampleMR package was used throughout. *p* < 0.05 was considered a statistically significant difference.

### 2.5 Retrospective study design

The diagnosis of ACS patients adheres to the ACS Emergency Rapid Diagnosis and Treatment Guidelines, which classify cases as STEMI, NSTEMI and UA (3). A total of 76 ACS patients admitted between April 1, 2022, and June 31, 2022, and who underwent coronary angiography at Shanghai East Hospital were included in our evaluation.

Inclusion criteria:

- Age ≥ 18 years old.
- Meets the ACS diagnostic criteria and is clearly diagnosed with ACS.

Exclusion criteria:

1. Patients who have previously been treated with PCSK9 inhibitors.
2. Persistent atrial fibrillation.
3. Severe aortic valve insufficiency or aortic valve stenosis.
4. Peripheral arterial disease indicated by an ankle-brachial index (ABI) of 0.9 or lower, or a history of lower limb bypass grafting and/or endovascular treatment.

There are two types of treatment for patients: receiving PCSK9 inhibitors (specifically, evolocumab 140 mg or alirocumab 75 mg) administered subcutaneously every 2 weeks in conjunction with statins (atorvastatin 20 mg or rosuvastatin 10 mg), as well as a control group receiving statins alone (atorvastatin 20 mg or rosuvastatin 10 mg). All participants were also administered a standard treatment regimen consisting of aspirin, clopidogrel/Tegrelo, angiotensin-converting enzyme inhibitor (ACEI) / adrenergic receptor binder (ARB), and β-blockers, unless contraindications were present for the use of these medications.

### 2.6 Clinical data collection

Patient demographics were gathered upon enrollment. Blood samples were obtained and AS indicators were evaluated before treatment initiation, as well as at 1 and 6 months post-treatment commencement. The collected blood samples were promptly transported to the laboratory at Shanghai East Hospital for analysis. Various indicators including troponin T (Tnt), N-terminal brain natriuretic peptide (NT-pro-BNP), total cholesterol (TC), triglyceride (TG), low-density lipoprotein (LDL), high-density lipoprotein (HDL), small dense low-density lipoprotein (sdLDL), lipoprotein (a)[Lp (a)], white blood cell count (WBC), neutrophil count (N), C-reactive protein (CRP), Interleukin-6 (IL-6), procalcitonin (PCT) and fibrinogen (FIB) were collected from all patients. To minimize bias, the same professional physician conducted measurements of right PWV (RPWV), left PWV (LPWV), right ABI (RABI) and left ABI (LABI) using the Omron Arteriosclerosis Tester (BP-203RPEIII) during a state of rest.

### 2.7 Cell culture and treatment

Mouse aortic smooth muscle cell lines (VSMCs, No. YC-A023) were purchased from Yuanjing Biotechnology (China, Guangzhou). The cells were cultured in DMEM/F12 (ShanghaiBasalmedia, China) medium containing 10% fetal bovine serum (Gibco, America) + 1% penicillin–streptomycin (NCMBiotech, China) in 5% CO_2_ at 37°C. The medium was changed every 2 days. According to published methods, 10 mmol/L β-gp, 20ug/m dexamethasone and 50ug/mL L-ascorbic acid were added to the routine medium to induce osteogenesis of VSMCs for 7 days, the medium were changed every 2/3 days (24–29). To mimic the effects of PCSK9i *in vivo*, 100 μg/mL of reagent-grade purified evolocumab (Selleck, America) was used to co-incubate with VSMCs for 7 days during osteogenesis induction (30–32). To mimic the stimulation of VSMCs by PCSK9 protein *in vivo*, refer to previous research (33), 0 μg/mL, 0.55 μg/mL, 1.1 μg/mL, 2.2 μg/mL, and 4.4 μg/mL of recombinant human PCSK9 (MedChemExpress, USA) were co-incubated with VSMCs for 7 days, and the concentration exhibiting the highest intracellular calcium content was chosen for subsequent experimental procedures.

### 2.8 Detection of calcium content within VSMCs

As per the guidelines provided by the Calcium Ion Assay Kit (Biyun Tian, China), the working solution for the assay was prepared by combining equal volumes of Solution A and Solution B. Subsequently, 50uL of VSMCs lysate was added to each well of the 96-well plate. Then 150uL of working solution per well was added to the 96-well plate and incubate for 10 min at room temperature, protected from light. Measure the absorbance at 575 nm to calculate the standard curve for calcium content. Analyze the remaining samples for BCA protein concentration. Express the Ca^2+^ concentration as calcium content divided by protein content in ug/mg.

### 2.9 Alizarin red staining

According to the instructions of the Alizarin Red Staining Kit (Biyun Tian, China), VSMCs were fixed with 4% paraformaldehyde for 30 min at room temperature. The cells were then washed with sterile PBS three times and incubated with Alizarin Red Staining Solution for 30 min at 37°C. After that, the cells were washed with ddH_2_O three times and observed under a 10X light microscope.

### 2.10 Alkaline phosphatase activity assay

According to the instructions of the Alkaline Phosphatase Assay Kit (Biyun Tian, China), prepare the substrate working solution by diluting the color-developing substrate. Then add 25uL of VSMCs lysate, 25uL of detection buffer, and 50uL of the chromogenic substrate to the sample wells of a 96-well plate. Incubate at 37°C for 10 min and stop the reaction by adding 100uL of termination solution to each well. Measure the absorbance at 405 nm. Express the results as the ratio of enzyme activity unit to protein concentration.

### 2.11 Western blot

VSMCs were lysed using RIPA buffer (ShanghaiEpizyme, China) containing a protease inhibitor mixture (ShanghaiEpizyme, China). After centrifugation, the supernatant was extracted. BCA kit (ShanghaiEpizyme, China) was used for protein concentration measurements with appropriate loading buffer. Extracted proteins (30 μg per lane) were run on an SDS-PAGE gel and then transferred to a PVDF membrane (Merck, Germany). Primary antibodies with *α*-SMA (arigobio, America) and runt-related transcription factor 2 (RUNX2, absin, China) were incubated overnight at 4°C. After careful washing with tween-containing triple-buffered saline (TBST), the membranes were incubated with horseradish peroxidase (HRP)-coupled secondary antibody (Biyun Tian, China, 1:2000) for 1 h at room temperature. The membranes were washed again three times for 10 min, and the signals were detected and quantified with Tanon 5,200 multifunctional image analysis system (Tanon Technology, Shanghai, China). The average gray values of the bands were analyzed utilizing Image J (NIH, Bethesda, USA) with GAPDH serving as the internal reference protein.

### 2.12 Statistical analysis

Data were analyzed using SPSS 25.0. Count data were described as frequencies (%). Continuous variables were expressed as mean ± standard deviation (SD) when normally distributed or median and interquartile range (IQR) when not normally distributed. Comparisons of intragroup continuous variables were performed using paired Wilcoxon rank sum tests. Comparisons of intergroup continuous variables were performed using independent sample *t*-tests for normally distributed data or Mann–Whitney *U*-tests for non-normal data. Comparisons for intergroup dichotomous variables were performed using chi-square or Fisher exact tests.

The Kolmogorov–Smirnov test was used to determine whether the distribution was normal or non-normal. The closeness test was analyzed using spearman correlation analysis. A *p*-value <0.05 was considered statistically significant.

## 3 Results

### 3.1 MR analysis reveals that genetic prediction of PCSK9 inhibition has potential to reduce the PWV

We identified 10 independent SNPs from the GWAS database (Supplementary Table S2). These SNPs are located within or near the PCSK9 gene, showing strong associations with PCSK9 and LDL-C levels in the GLCC dataset. Consequently, these SNPs can serve as genetic proxies for PCSK9 and imitate the effects of PCSK9i. To ensure the exclusion of confounding factors, we employed PhenoScanner, heterogeneity analyses, multiplicity analyses, MR-PRESSO and leave-one-out tests to establish the relationship between these SNPs and the outcomes (Supplementary Table S3).

**Figure 2:**
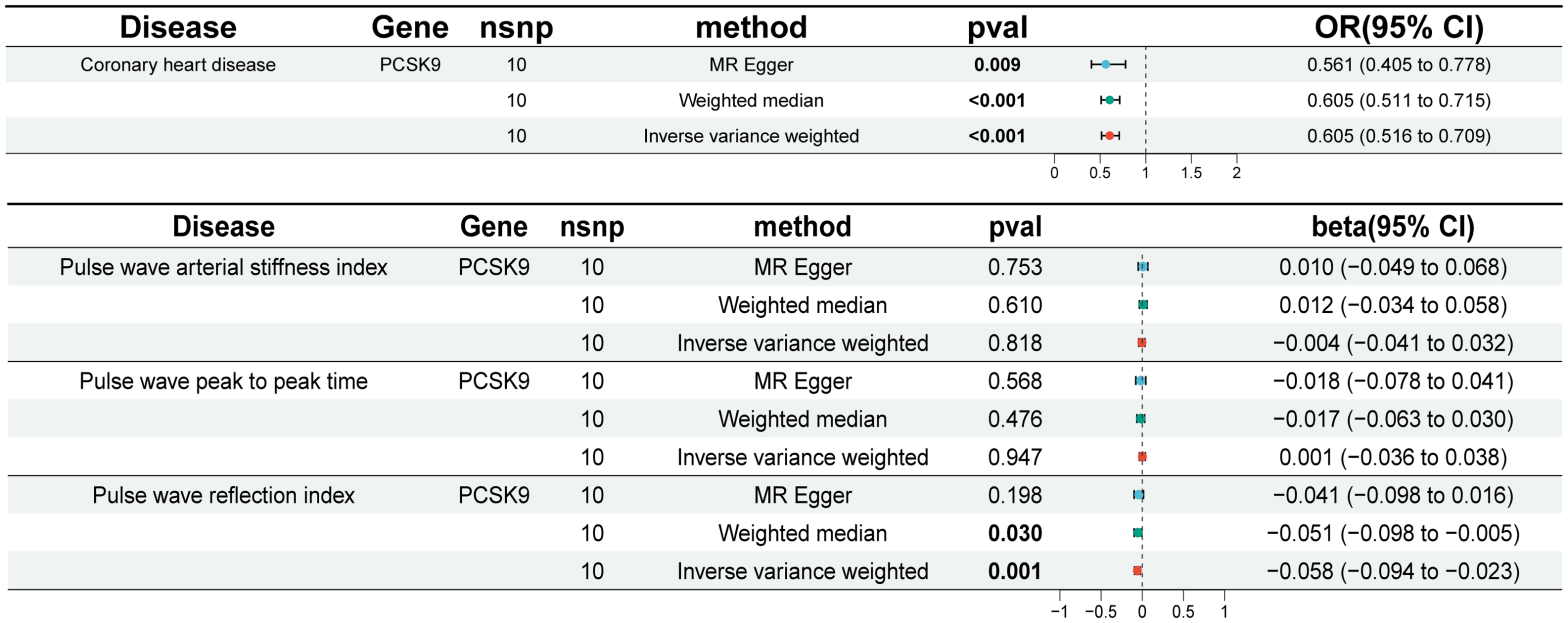
Results of MR analysis. CI, Confidence Interval.

Given the widespread utilization of PCSK9i in the treatment of CHD, we employed aggregated data from the coronary GWAS as a means of confirming the reliability of the SNPs. The implementation of the IVW method yielded statistically significant evidence (OR [95%] = 0.605 [0.516 ~ 0.709], *p* = 0.001) showcasing a noteworthy decrease in the risk of CHD through the use of PCSK9i (Figure 2). This outcome was consistently observed across both the weighted median and MR Egger approaches. The validation of the positive control further substantiates credibility of the SNPs.

In our study, we utilized the IVW method to conduct correlation analysis. Our findings indicate a negative correlation between genetic prediction of PCSK9 inhibition and PWRI (beta [95% CI] = −0.058 [−0.094 ~ −0.023], *p* = 0.001). However, we did not observe significant correlations with PWPPT and PWASI (Figure 2). Sensitivity analysis revealed no evidence of heterogeneity or horizontal pleiotropy across all outcomes (*p* > 0.05). Additionally, the retention method demonstrated that removing SNPs did not significantly impact the results. Cochran’s Q-test did not identify any indications of heterogeneity (Supplementary Table S3). The leave-one-out method demonstrated no statistically significant variance in the results following the exclusion of SNPs (Supplementary Figure 1).

### 3.2 Retrospective study indicates that PCSK9i effectively ameliorate blood lipids, inflammatory factors and PWV in ACS patients

**Figure 3:**
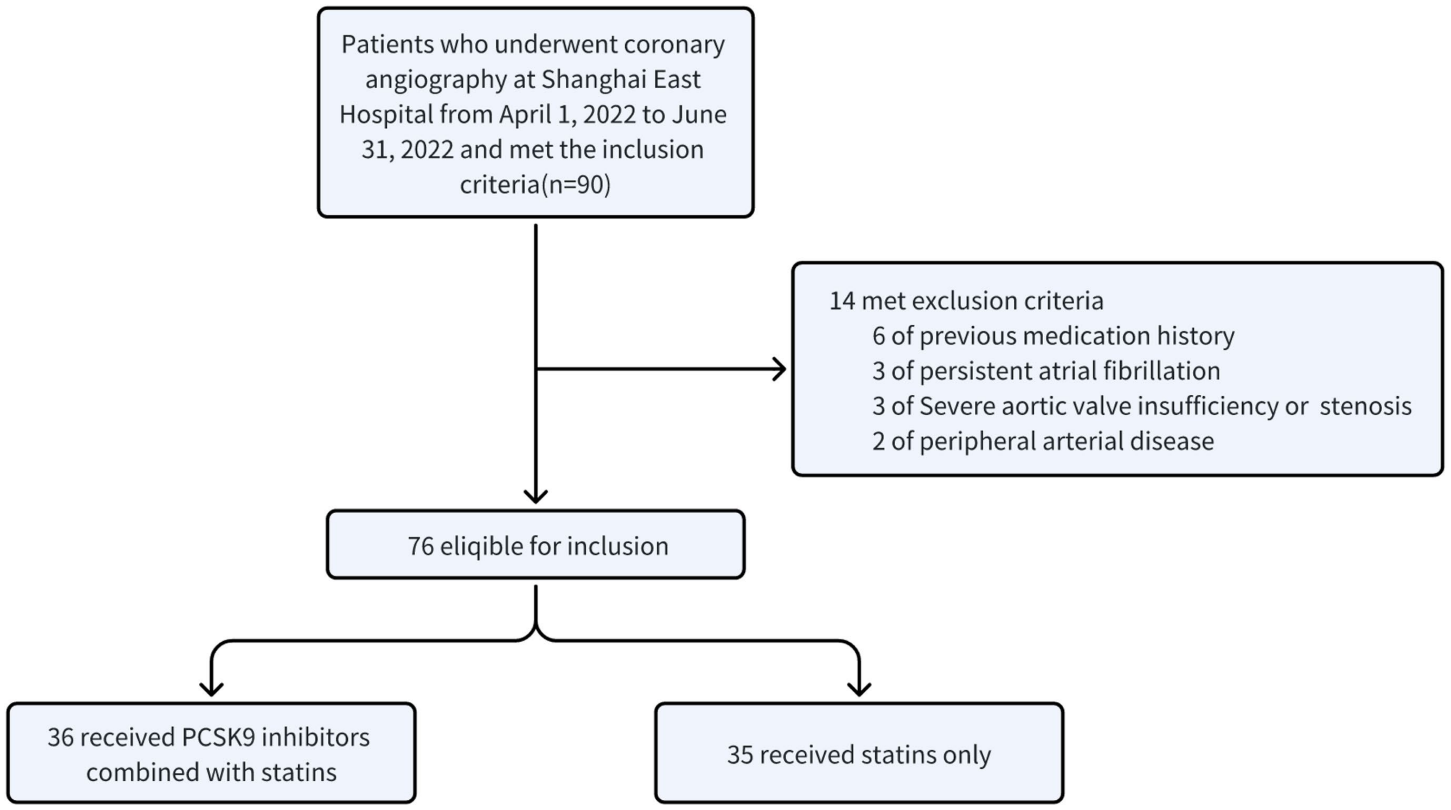
Flow chart of inclusion and exclusion.

A total of 90 patients with ACS after PCI were diagnosed as ACS. Ultimately, 71 eligible patients included the PCSK9i group (*n* = 36) and the control group (*n* = 35) were retrospectively collected (Figure 3). There were no notable disparities observed in the overall data of the two groups, encompassing fundamental information, medical history, and medication history (Table 1, *p* > 0.05). However, discrepancies were identified solely in smoking history (Table 1, *p* = 0.013) and the administration of β-blocker (Table 1, *p* = 0.001). Moreover, no statistically significant variances were found in baseline data, CRP, FIB, IL-6, PCT, or lipid levels between the two groups (Table 2, *p* > 0.05).

**Table 1:** Clinical characteristics for ACS study group (*n* = 71).

Clinical characteristics	Total (*n* = 71)	PCSK9i group (*n* = 36)	Control group (*n* = 35)	*p*-value
Age (years)	66.8 ± 11.2	66 (36–91)	71 (49–88)	0.247
Men, *n* (%)	52 (73.2)	28 (77.8)	24 (68.6)	0.43
BMI (kg × m^−2^)	25.02	24.6 (19.3–31.2)	25.1 (19–30.8)	0.739
Coronary heart disease, *n* (%)	12 (16.9)	7 (19.4)	5 (14.3)	0.562
Diabetes mellitus, *n* (%)	18 (25.4)	6 (16.7)	12 (34.3)	0.088
Heart failure, *n* (%)	4 (5.6)	2 (5.6)	2 (5.7)	0.977
Stroke history, *n* (%)	2 (2.8)	1 (2.8)	1 (2.9)	0.984
Hypertension, *n* (%)	40 (56.3)	23 (63.9)	17 (48.6)	0.193
Smoking history, *n* (%)	35 (49.3)	23 (63.9)	12 (34.3)	0.013**
STEMI, *n* (%)	46 (64.8)	27 (75)	19 (54.3)	0.08
NSTEMI, *n* (%)	15 (21.1)	7 (19.4)	8 (22.9)	0.08
Unstable angina, *n* (%)	10 (14.1)	2 (5.6)	8 (22.9)	0.08
ARB, *n* (%)	58 (81.7)	31 (81.6)	27 (73)	0.329
ACEI, *n* (%)	3 (4.2)	2 (5.3)	1 (2.7)	0.572
*β*-blocker, *n* (%)	61 (85.9)	36 (94.7)	25 (67.6)	0.001**
CCB, *n* (%)	18 (25.4)	7 (18.4)	11 (29.7)	0.246
Diuretic, *n* (%)	34 (47.9)	18 (47.4)	16 (43.2)	0.718
Hypoglycemic drugs, *n* (%)	11 (15.5)	7 (18.4)	4 (10.8)	0.351
Anticoagulation, *n* (%)	41 (57.7)	22 (57.9)	19 (51.4)	0.561

*p < 0.05, **p < 0.01.

**Table 2:** Clinical feature of the study population at baseline.

Clinical characteristics	PCSK9i group (*n* = 36)	Control group (*n* = 35)	*p*-value
Tnt (ng/ml)	1.23 (0.005–10)	1.38 (0.006–10)	0.284
FBG (mmol/L)	5.7 (3.74–18.97)	6.33 (3.97–16.4)	0.252
TC (mmol/L)	4.46 (3.13–9.05)	4.38 (2.05–8.23)	0.243
TG (mmol/L)	1.41 (0.65–3.82)	1.53 (0.65–3.5)	0.421
HDL (mmol/L)	1.02 (0.63–2)	1.01 (0.67–1.74)	0.904
LDL (mmol/L)	2.96 (1.56–6.91)	2.76 (1.76–5.69)	0.476
sdLDL (mmol/L)	0.84 (0.33–1.98)	0.63 (0.2–1.98)	0.531
Lp(a) (mmol/L)	37 (1–257)	25 (4–235)	0.272
WBC (*10^9/L)	10.09 (6–17.86)	9.12 (5.77–17.39)	0.178
N (*10^9/L)	7.91 (3.11–12.19)	6.99 (3.09–12.45)	0.501
CRP (mg/L)	2.03 (1.6–85.53)	2.3 (1.6–104.05)	0.218
FIB (g/L)	3.13 (1.77–6.77)	3.61 (2.06–7.54)	0.051
IL-6 (pg/ml)	14.68 (1.73–91.94)	15.71 (3.22–50.01)	0.565
PCT (ng/ml)	0.207 (0.02–0.677)	0.32 (0.013–4.65)	0.242

In comparison to the baseline data, the PCSK9i group exhibited a statistically significant and persistent reduction in the levels of inflammatory markers WBC, N, CRP, IL-6 and PCT at both 1 and 6 months. Additionally, the levels of TC, LDL, Lp(a), and sdLDL continued to decrease significantly. However, there was no significant decrease in TG levels at 1 month, with the results lacking statistical significance. Nevertheless, a statistically significant decrease was observed at 6 months. Furthermore, the patients’ HDL levels displayed a tendency to increase at both 1 month and 6 months, with statistically significant results (Table 3).

**Table 3:** Indicators of the PCSK9i group after 1 month and 6 months.

Indicators	Baseline	1 month	6 months	*p*-value (1 month vs. Baseline)	*p*-value (6 months vs. 1 month)
TC (mmol/L)	4.46 (3.13–9.05)	3.47 (1.79–4.9)	2.778 (1.64–4.21)	0.000**	0.000**
TG (mmol/L)	1.41 (0.65–3.82)	1.31 (0.72–2.89)	1.38 (0.70–2.32)	0.060	0.003**
HDL (mmol/L)	1.02 (0.63–2)	1.07 (0.83–1.92)	1.21 (0.73–1.82)	0.000**	0.000**
LDL (mmol/L)	2.96 (1.56–6.91)	1.89 (0.34–3.12)	1.4 (0.43–2.27)	0.000**	0.000**
sdLDL (mmol/L)	0.84 (0.33–1.98)	0.77 (0.28–1.99)	0.72 (0.13–1.76)	0.017*	0.003**
Lp (a) (mmol/L)	37 (1–257)	32 (2–162)	31.5 (4–87)	0.003**	0.231
WBC (*10^9/L)	10.09 (6–17.86)	8.29 (5.12–11.17)	7.88 (5.83–9.67)	0.000**	0.157
N (*10^9/L)	7.91 (3.11–12.19)	5.33 (2.91–8.23)	5.35 (3.14–6.95)	0.000**	0.610
CRP (mg/L)	2.03 (1.6–85.53)	1.6 (1.6–7.92)	1.6 (1.21–5.1)	0.000**	0.968
FIB (g/L)	3.13 (1.77–6.77)	2.23 (1.17–4.27)	1.99 (1.21–3.32)	0.000**	0.003**
IL-6 (pg/ml)	14.68 (1.73–91.94)	7.3 (1.71–21.77)	4.18 (1.17–7.3)	0.000**	0.000**
PCT (ng/ml)	0.207 (0.020–0.677)	0.095 (0.010–0.350)	0.160 (0.010–0.320)	0.000**	0.030*

*p < 0.05, **p < 0.01.

In comparison to the baseline data, the control group exhibited a statistically significant reduction in inflammatory factors WBC, N, CRP, IL-6, and PCT following 1 month of treatment. Nonetheless, the extended duration of treatment to 6 months did not yield any additional improvements in the remaining markers, including IL-6. Lp(a) and sdLDL did not exhibit significant changes, and the observed results did not demonstrate statistical significance. Conversely, the patients’ TC and LDL continued to decrease in a statistically significant manner. Notably, HDL did not display significant changes at the 1-month checkpoint, and these findings also lacked statistical significance. However, a noteworthy increase was observed at the conclusion of the 6-month follow-up period, with the results demonstrating statistical significance (Table 4).

**Table 4:** Indicators of the control group after 1 month and 6 months.

Indicators	Baseline	1 month	6 months	*p*-value (1 month vs. Baseline)	*p*-value (6 months vs. 1 month)
TC (mmol/L)	4.38 (2.05–8.23)	3.92 (2.15–5.95)	3.76 (2.56–5.13)	0.002**	0.035*
TG (mmol/L)	1.53 (0.65–3.5)	1.40 (0.49–3.2)	1.38 (0.57–3.06)	0.027*	0.147
HDL (mmol/L)	1.01 (0.67–1.74)	1.09 (0.73–1.75)	1.12 (0.72–1.68)	1.000	0.001**
LDL (mmol/L)	2.76 (1.76–5.69)	2.10 (1.33–3.66)	1.99 (0.76–3.17)	0.000**	0.012*
sdLDL (mmol/L)	0.63 (0.2–1.98)	0.75 (0.32–1.85)	0.77 (0.29–1.82)	0.161	0.539
Lp (a) (mmol/L)	25 (4–235)	36 (7–217)	33 (7–116)	0.752	0.959
WBC (*10^9/L)	9.12 (5.77–17.39)	8.01 (4.27–9.82)	7.99 (5.23–9.79)	0.000**	0.164
N (*10^9/L)	6.99 (3.09–12.45)	5.71 (2.16–7.36)	5.87 (3.34–7.55)	0.000**	0.408
CRP (mg/L)	2.3 (1.6–104.05)	4.99 (1.6–68.8)	5.47 (1.6–29.4)	0.050	0.265
FIB (g/L)	3.61 (2.06–7.54)	3.07 (1.97–7.32)	3.01 (1.66–5.79)	0.013*	0.322
IL-6 (pg/ml)	15.71 (3.22–50.01)	9.76 (2.2–99)	5.89 (1.65–20.1)	0.001**	0.001**
PCT (ng/ml)	0.32 (0.013–4.65)	0.178 (0.010–0.83)	0.170 (0.010–1.35)	0.012*	0.459

*p < 0.05, **p < 0.01.

**Figure 4:**
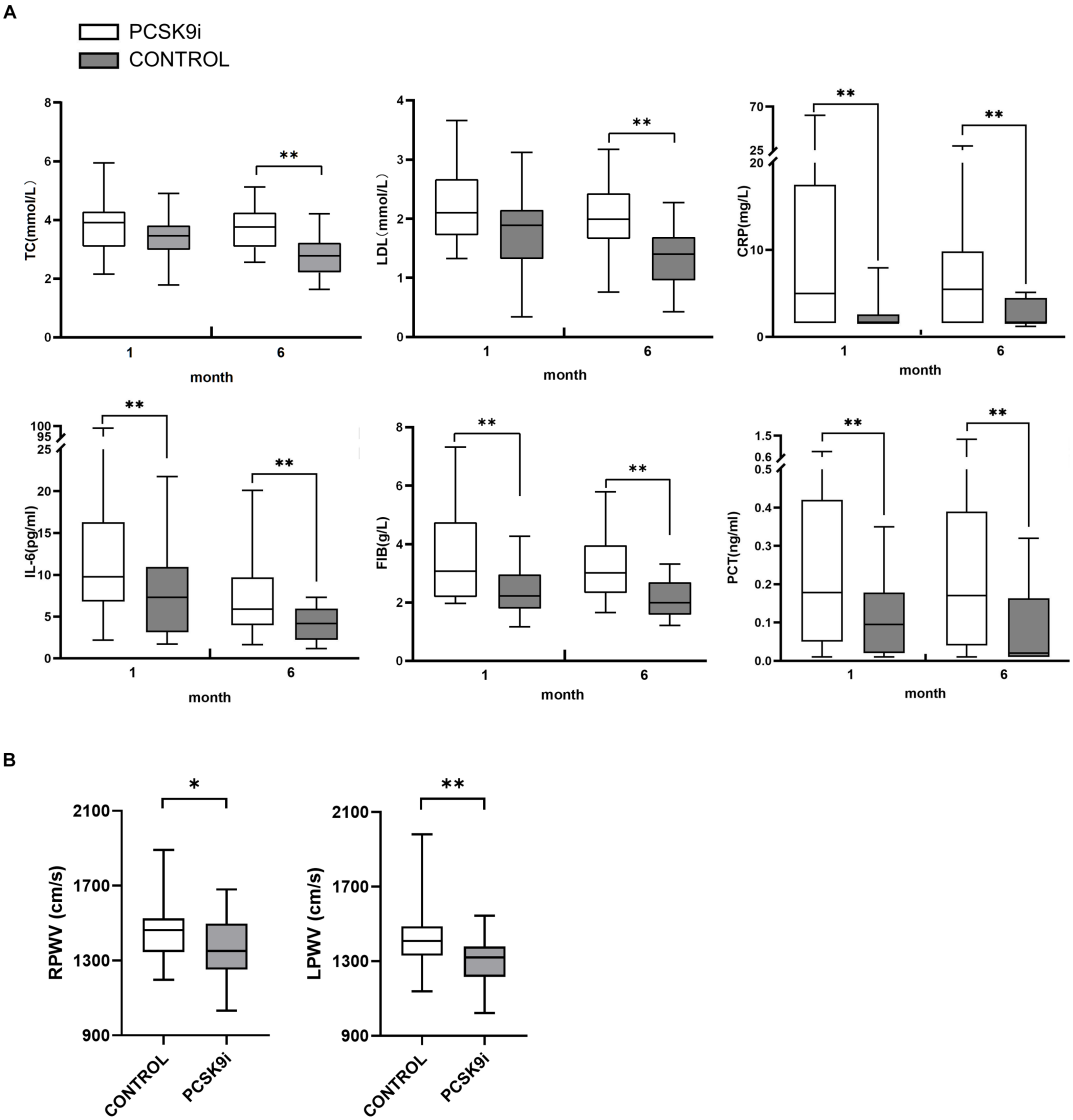
**(A)** The differences of TC, LDL-C, CRP, FIB, IL-6 and PCT in 2 groups at 1 and 6 months of treatment **(B)** The differences of RPWV and LPWV in 2 groups at 1 and 6 months of treatment (*p < 0.05,**p < 0.01).

In comparison to the control group, the PCSK9i group exhibited statistically significant enhancements in CRP, FIB, IL-6 and PCT following a 1-month intervention. Nevertheless, there was no notable disparity in lipid levels between the two groups. Subsequent to 6 months of treatment, there was a statistically significant reduction in TC, LDL-C, CRP, FIB, IL-6 and PCT levels (Figure 4A).

At the baseline level, there was no statistically significant difference between PWV and ABI between the two groups (*p* > 0.05). After 1 month of treatment, RPWV (*p* < 0.05) and bilateral ABI (*p* < 0.01) were reduced in the PCSK9i group, and the difference in LPWV was not statistically significant compared with baseline (*p* > 0.05), and no significant changes in PWV and ABI were observed in the control group (*p* > 0.05). After 6 months of treatment, there was a significant decrease in both PWV and ABI in the PCSK9i group (*p* < 0.01) compared to 1 month. In comparison, there was no significant change in PWV and ABI in the control group (*p* > 0.05) (Table 5).

**Table 5:** PWV of the two groups after 1 month and 6 months.

Group	Indicators	Baseline	1 month	6 months	*p* –Value (1 month vs. Baseline)	*p* –Value (6 months vs. 1 month)
PCSK9i group	LPWV (cm/s)	1448.5 (1106–1796)	1,467 (1098–1791)	1,320 (1021–1,543)	0.219	<0.001**
	RPWV (cm/s)	1497.5 (1133–1912)	1488.5 (1103–1882)	1,350 (1032–1,679)	0.026*	<0.001**
	LABI	1.07 (1–1.11)	1.04 (1.01–1.1)	1.02 (0.99–1.06)	0.001**	<0.001**
	RABI	1.03 (0.99–1.1)	1.03 (1–1.07)	1.01 (0.98–1.03)	0.002**	<0.001**
Control group	LPWV (cm/s)	1,405 (1128–2013)	1,405 (1138–1987)	1,407 (1138–1980)	0.849	0.850
	RPWV (cm/s)	1,451 (1176–1873)	1,461 (1185–1882)	1,465 (1196–1890)	0.238	0.702
	LABI	1.03 (0.9–1.1)	1.04 (0.92–1.09)	1.06 (0.94–1.09)	0.358	0.680
	RABI	1.02 (0.91–1.1)	1.04 (0.91–1.09)	1.04 (0.95–1.09)	0.888	0.158
*p*-value (PCSK9i vs. Control)	LPWV (cm/s)	0.792	0.980	<0.001**	——	——
	RPWV (cm/s)	0.413	0.423	0.012*	——	——
	LABI	0.775	0.629	0.152	——	——
	RABI	0.580	0.791	0.089	——	——

*p < 0.05, **p < 0.01.

Comparing the AS parameters between the two groups of patients after 6 months of treatment, there was no significant difference in ABI (*p* > 0.05), but PWV in the PCSK9i group was significantly lower than that in the control group (*p* < 0.05) (Table 5 and Figure 4B).

A correlation analysis was conducted between the changes in blood lipids and inflammatory factors in the PCSK9i group after 6 months of treatment and the changes in PWV. Only ΔCRP and ΔPWV were correlated (Table 6, *r* = 0.343, *p* = 0.043).

**Table 6:** The relationship between ΔPWV and changes in inflammatory factors and blood lipids.

Indicators	*r -*value	*p -*value
ΔTC	0.088	0.615
ΔTG	0.026	0.883
ΔHDL	0.289	0.093
ΔLDL	0.152	0.382
ΔsdLDL	0.198	0.255
ΔLp(a)	0.190	0.275
ΔWBC	0.110	0.528
ΔN	0.194	0.265
ΔCRP	0.343	0.043*
ΔFIB	0.281	0.103
ΔIL-6	0.125	0.473
ΔPCT	0.240	0.164

*p < 0.05.

### 3.3 Cell experiments indicate that PCSK9i ameliorates osteogenesis of VSMCs

**Figure 5:**
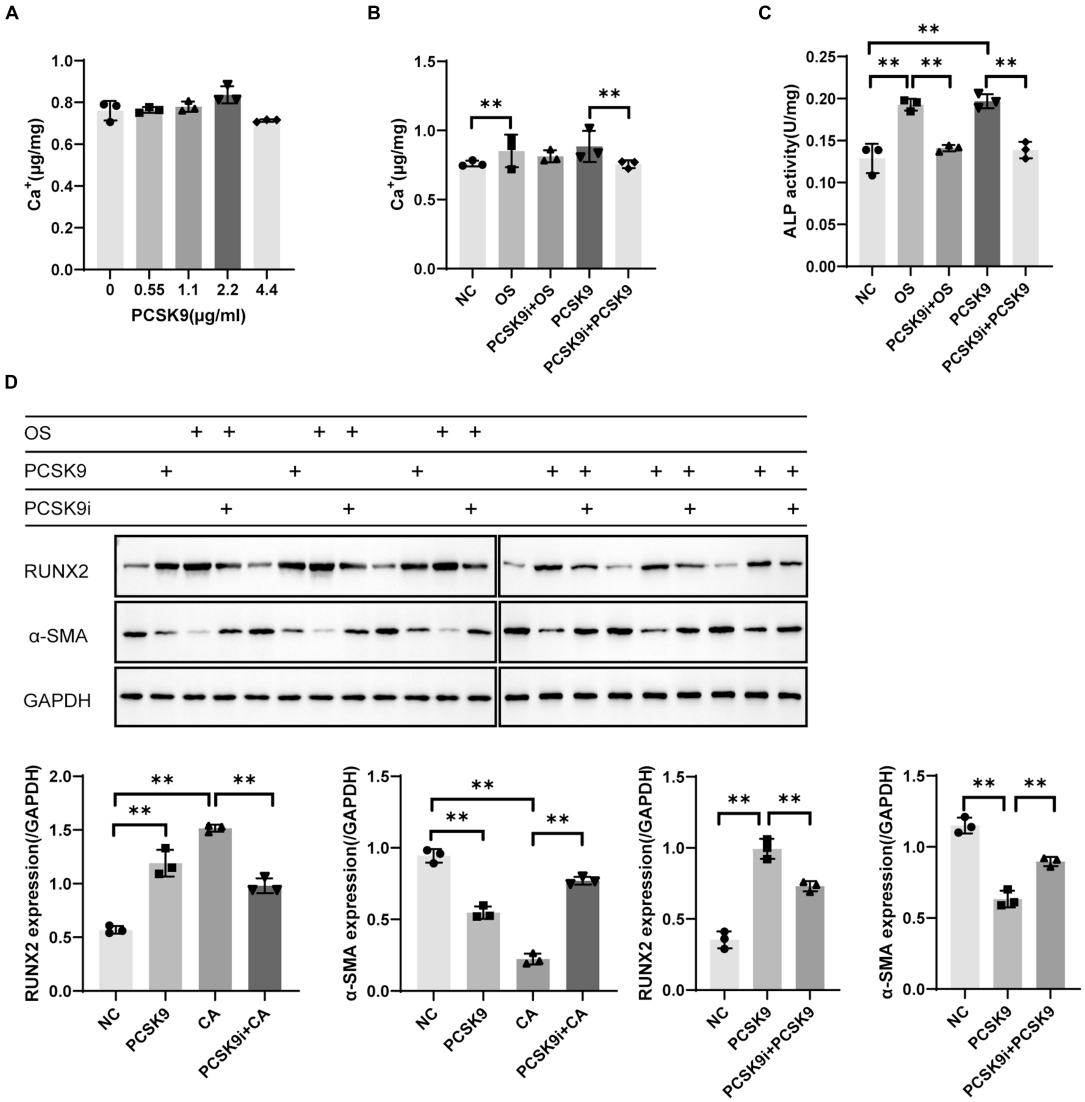
**(A)** Calcium content in VSMCs with different concentration of PCSK9 co-incubated **(B)** Calcium content in VSMCs of different groups **(C)** ALP activity in VSMCs of different groups **(D)** RUNX2 and *α*-SMA expression in VSMCs of different groups. **p < 0.01, NC, normal control; OS, osteogenesis; PCSK9i + OS, PCSK9i & osteogenesis; PCSK9, PCSK9 protein; PCSK9i + PCSK9, PCSK9i & PCSK9 protein.

In the pre-experiment we found that the intracellular calcium content showed a concentration-dependent increase with increasing PCSK9 concentration. The highest intracellular calcium content of 2.2 μg/mL PCSK9 was selected for subsequent experiments (Figure 5A). The intracellular calcium content increased significantly in the osteogenic group (OS) compared to the control group (NC) and decreased after treatment with PCSK9i compared to OS. After stimulating the cells with PCSK9, the intracellular calcium content increased compared to NC. After treatment with PCSK9i, the intracellular calcium content decreased compared to the PCSK9 group (Figure 5B).

**Figure 6:**
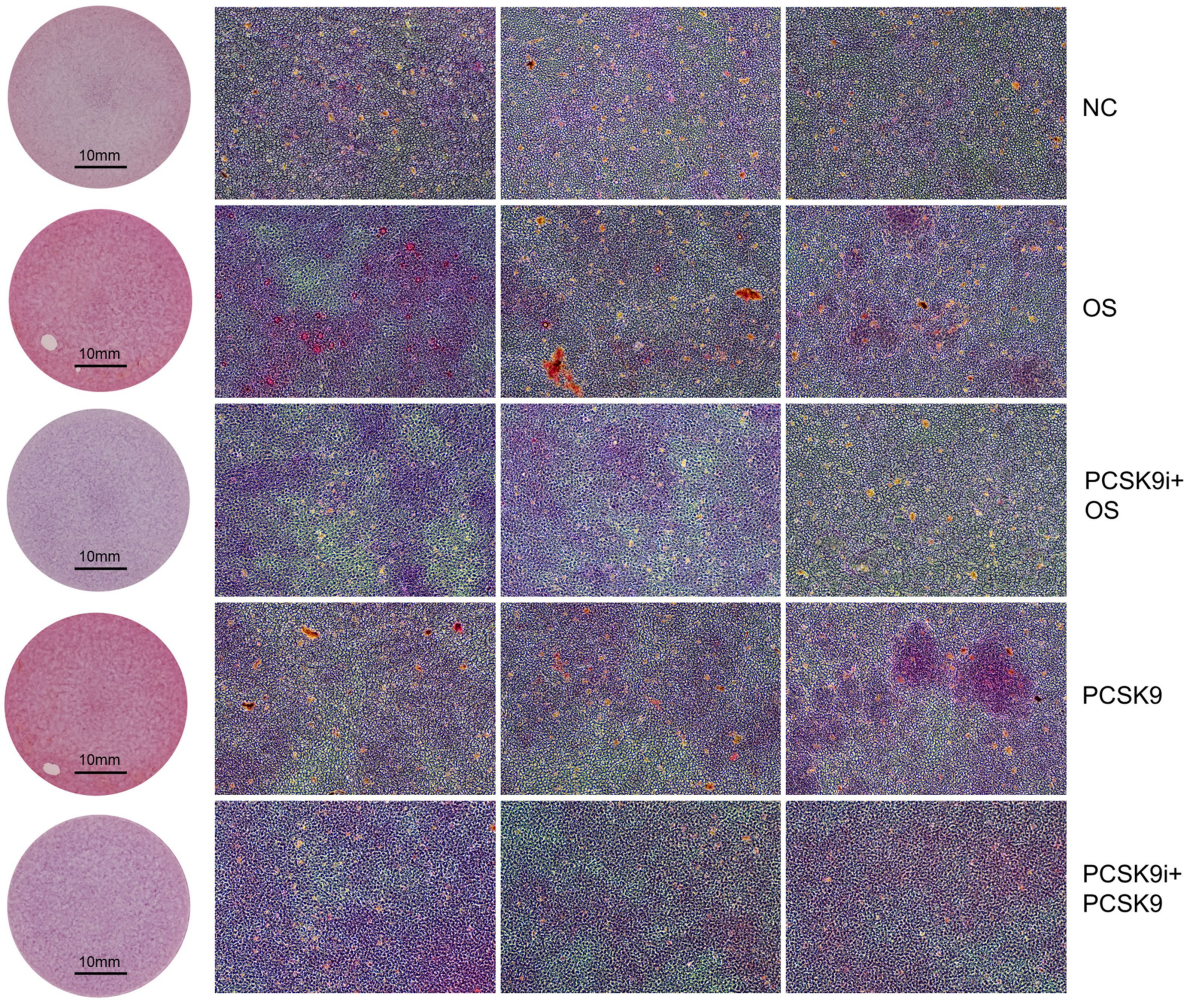
Calcium deposition was visualized using Alizarin red staining (reddish signal). Representative macroscopic (circular field of view) and microscopic (square field of view, 10X). NC, normal control; OS, osteogenesis; PCSK9i + OS, PCSK9i & osteogenesis; PCSK9, PCSK9 protein; PCSK9i + PCSK9, PCSK9i & PCSK9 protein.

In this experiment, an increase in the intensity of red hues correlates with a higher concentration of calcium salt deposition. Calcium salt deposition was significantly increased in OS compared to NC and was significantly decreased after treatment with PCSK9i compared to OS. After stimulation of cells with PCSK9, calcium salt deposition increased compared to NC. After treatment with PCSK9i, calcium salt deposition was decreased compared to PCSK9 group (Figure 6).

ALP activity was significantly increased in OS compared to NC and significantly decreased after treatment with PCSK9i compared to OS. After stimulation of cells with PCSK9, ALP activity increased compared to NC. After treatment with PCSK9i, ALP activity was decreased compared to PCSK9 group (Figure 5C).

WB results showed that RUNX2 expression was up-regulated and *α*-SMA expression was down-regulated in OS compared to NC, and RUNX2 expression was down-regulated and *α*-SMA expression was up-regulated after the treatment with PCSK9i compared to OS. After stimulation of cells with PCSK9, RUNX2 expression was up-regulated and *α*-SMA expression was down-regulated compared to NC. After treatment with PCSK9 inhibitor, RUNX2 expression was down-regulated and *α*-SMA expression was up-regulated compared to PCSK9 group (Figure 5D).

## 4 Discussion

Due to the multifaceted characteristics of PCSK9i and the significant involvement of AS in the pathogenesis of cardiovascular disease, our objective was to investigate the potential association between PCSK9i and AS in individuals diagnosed with ACS.

Initially, a two-sample MR analysis was conducted. Considering the established impact of PCSK9 inhibition on LDL levels (34), the selection of variants resembling PCSK9 proxies near the PCSK9 gene was based on LDL levels as a criterion. The utilization of a drug-targeted MR method has been extensively employed in prior published studies (18, 20, 35–37). In order to enhance the credibility of these SNPs, we employed the CHDdataset as a positive control and observed a significant decrease in risk of CHD associated with these SNPs, thus validating their suitability as IVs. Based on our findings, it can be inferred that genetically predicted PCSK9 inhibition exhibits a negative correlation with AS parameter PWRI. Consequently, we propose the hypothesis that PCSK9i has the potential to mitigate AS. Similar results have been described in several clinical studies, but no evidence has been published at the genome-wide level, which is the innovation of this study.

The relationship between PCSK9i and AS has been documented in a subset of clinical studies involving patients with FH. Ruscica et al. conducted a comprehensive randomized clinical trial, which demonstrated a noteworthy correlation between circulating PCSK9 and PWV. Furthermore, the use of a PCSK9i to decrease circulating PCSK9 levels may have a beneficial impact on the amelioration of AS (14). Papaioannou et al. observed a notable enhancement in PWV subsequent to the incorporation of PCSK9i into statin therapy among individuals with FH (38). Scicali R et al. found that PCSK9i significantly reduced PWV in patients with FH after 6 months of treatment (39) and that lowering LDL-C was associated with improved PWV. However, there exist notable distinctions between patients with ACS or FH in relation to their internal environment, pathophysiologic mechanisms and interventions. At present, there is a dearth of research examining whether patients with ACS experience equivalent AS - reducing advantages from PCSK9i.

Therefore, we proceeded to conduct a retrospective study in order to examine the alterations in lipids, inflammatory markers, and AS among patients with ACS following the administration of statins and PCSK9i. We uncovered significant insights indicating that smoking significantly increases PWV (40). Despite a higher proportion of smokers in the PCSK9i group, our results surprisingly demonstrate a greater reduction in PWV within this cohort. Additionally, findings from another study indicate that differences in baseline β-blocker usage do not have a significant impact on PWV (41). Therefore, we believe that these differences between the two groups will not affect the final conclusion. Consistent with numerous contemporary clinical investigations, both PCSK9i and statins exhibited noteworthy lipid-lowering effects in our cohort. However, these effects varied with respect to lipid compositions. After 6 months of treatment, patients in PCSK9i group showed significant decreases in TC, TG, LDL, sdLDL, and Lp (a), while HDL increased significantly. In control group using statins we similarly observed improvements in TC, TG, LDL, and HDL, but not in Lp(a) and sdLDL.

We chose PWV which is currently considered the “gold standard” in clinical practice as the primary measure to evaluate AS in patients with ACS. Additionally, we utilized ABI as a supplementary measure representing the ratio of ankle arterial pressure to brachial arterial pressure. ABI serves as an indicator for the presence of atherosclerosis or stenosis in the lower extremities (42). Based on data from several studies (43). ABI <0.90 has been widely accepted as a diagnostic criterion for lower extremity arterial disease, and the PWV data of such patients with severe atherosclerosis or stenosis of the lower extremity arteries could not reflect the true AS, so we used ABI <0.9 as an exclusion criterion for the study design. Concurrently, considering the association between ABI and AS, along with ABI’s predictive capability for adverse cardiovascular events (44–46), we incorporated ABI as an additional component in our analysis to achieve a more comprehensive evaluation of AS.

There was an absence of disparity in baseline PWV between the PCSK9i and control groups. Following 1 month of treatment, a decrease in RPWV was observed in the PCSK9i group, although no statistically significant variation in PWV was evident between the two groups. However, after 6 months of treatment, PWV was significantly lower in the PCSK9i group compared to 1 month, which was not observed in control group. PWV was significantly lower in the PCSK9i group than in the control group after 6 months of treatment. These data suggest that PCSK9i are effective in improving AS in patients with ACS, but may require a longer period of time to demonstrate a significant effect. This finding is similar to that of Papaioannou, Roberto. S, and others, who also found that a reduction in LDL-C was associated with improved PWV in patients with familial hypercholesterolemia (39). However, by correlating ΔPWV with ΔLDL in PCSK9i group, we did not reach the same conclusion. The reason for this situation may be due to the difference in the study population. The previous study focused on patients with FH, while our study focused on patients with ACS. Due to the distinct characteristics of these diseases, improving AS in ACS patients with PCSK9i may not solely be achieved by reducing LDL levels.

Lp(a) is an LDL particle bound to apolipoprotein (a), which carries oxidized phospholipids that adversely affect a variety of pathways including inflammation, endothelial function, and thrombosis (47). Lp (a) is also known to contribute to the progression of atherosclerosis and increase the risk of atherosclerotic cardiovascular disease (ASCVD) (48). High levels of Lp(a) have been linked to a higher risk of diseases like CVD and stroke as indicated by multiple studies (48–50). Several clinical trials have been conducted to examine the efficacy of PCSK9 inhibitor therapy in reducing Lp(a) and sdLDL (47, 51, 52). Our cohort study also showed significant reductions in Lp (a) and sdLDL levels in patients treated with PCSK9i, indicating that PCSK9i may target these two lipid components. However, correlation analyses did not find a link between ΔPWV and reductions in these lipoproteins, suggesting that PCSK9i may improve AS through other mechanisms.

The conventional perspective posits that lipids are the primary etiological factor in CHD. Despite the general populace has experienced a consistent decline in total plasma cholesterol levels because of the ongoing advancements in lipid-lowering medications (53–55), nevertheless, a notable prevalence of ASCVD events persists, even among individuals who have effectively managed their LDL levels (56). As lipids alone proved insufficient in accounting for all occurrences of ASCVD, subsequent investigations gradually revealed additional potential mechanisms, with inflammation emerging as a particularly significant factor (57). Crea et al. summarized the possible mechanisms that predispose to ACS, suggesting that myocardial infarction (MI) may result from plaque rupture triggered by a systemic inflammatory response (58). In addition, about 60% of patients with MI have a high initial CRP (≥2.0 mg/L) (59). In the context of MI, the process of cardiomyocyte necrosis results in the release of harmful molecules that interact with pattern-recognition receptors, such as toll-like receptors. This interaction combined with complement activation and the presence of reactive oxygen species, leads to the upregulation of cytokines and chemokines. Consequently, these molecular events contribute to the development of both coronary and systemic inflammatory responses (60). This inflammatory response has the potential to inflict additional harm upon the myocardium, resulting in heart failure, inducing a pervasive inflammation across the coronary arteries, heightening the likelihood of recurring myocardial infarction, and ultimately contributing to elevated post-infarction mortality rates (61). However, due to the lack of efficacy demonstrated in multiple trials focusing on the early inflammatory response following ACS (62), the majority of clinical guidelines do not endorse the use of anti-inflammatory therapy for patients diagnosed with ACS. However, in clinical practice we have observed that ACS patients with PCSK9i tend to have more significant improvements in inflammatory factors, even if they do not receive anti-inflammatory therapy. PCSK9i may have anti-inflammatory effects due to its close association with inflammatory processes (63, 64). Therefore, we also examined and analyzed changes in commonly observed inflammatory factors in this study.

Compared to the baseline, inflammatory factors WBC, N, CRP, IL-6, and PCT consistently decreased in the PCSK9i group at 1 and 6 months. The control group also showed significant decreases in these factors after 1 month, but no further improvement was observed except for IL-6 at 6 months. After 1 and 6 months of treatment, PCSK9i group showed significantly lower levels of CRP, IL-6, and PCT compared to control group. However, there were no significant differences in WBC and N between two groups. All ACS patients we studied were discharged from the hospital with improved conditions after treatment. Therefore, the improvement of inflammatory markers in both groups after 1 month of treatment does not necessarily indicate the anti-inflammatory effects of PCSK9i or statins. It is possible that these inflammatory factors naturally reduce as the disease state improves. However, with continued use of the drug, patients in PCSK9i group demonstrated a sustained decrease in the 3 inflammatory markers after 6 months which was difficult to be explained by the self-limiting nature of the disease. In contrast, control group did not observe the same changes. After 6 months of treatment, the PCSK9i group showed significantly lower levels of the 3 inflammatory factors compared to control group, highlighting the distinct anti-inflammatory impact of PCSK9i in contrast to statins.

In addition, we conducted a correlation analysis between alterations in inflammatory factors and ΔPWV after 6 month in PCSK9i group. Interestingly, our findings revealed that only ΔCRP exhibited a significant correlation with ΔPWV. C-reactive protein, a cyclic pentameric glycoprotein predominantly synthesized in the liver, has been extensively investigated in the context of cardiovascular disease. A substantial body of evidence substantiates the utility of CRP as a guide for therapeutic interventions in primary prevention (65). A meta-analysis that included more than 160,000 individuals with new-onset ASCVD showed that increased hsCRP levels were associated with an increased risk of CHD, ischemic stroke, and vascular mortality (66). The JUPITER trial demonstrated that daily administration of Rosuvastatin significantly reduced the incidence of first MI, stroke, or cardiovascular death in individuals with hsCRP ≥2 mg/L and LDL-C < 130 mg/dL (67). Based on these observations, the guidelines for blood cholesterol management encourage consideration of statins in patients with hsCRP ≥2 mg/L (68). While CRP’s connection to ASCVD has been extensively researched, its association with AS remains understudied. In patients with hyperlipidemia, there was a significant positive correlation between AS and CRP, with correlations ranging from mild to moderate associations (Pearson *r* = 0.33 to *r* = 0.624) (69). In atherosclerotic population, higher CRP levels at baseline were independently associated with reduced aortic distensibility (70). Previous studies have primarily examined the link between CRP levels and AS, but rarely explored the connection between lower CRP and improved PWV. Our study discovered that lower CRP levels were associated with improved PWV in ACS patients, and this reduction in CRP was due to the anti-inflammatory effects of PCSK9i.

Although our spearman correlation analysis only shows a correlation between ΔCRP and ΔPWV but cannot determine causality, our data suggest that CRP levels decrease before significant changes in PWV occur after 1 month of treatment. This indicates that improving CRP levels is prioritized over improving PWV. Therefore, we speculate that the potential mechanism by which PCSK9i improve AS may be partially related to reducing CRP. Further studies are needed to confirm this, and we eagerly await future research results.

We further designed cellular experiments to validate the effect of PCSK9i on AS. Osteogenesis of VSMCs is one of the main response indicators of AS at the cellular level. The expression of RUNX2 plays a key role in the osteogenesis of VSMCs. In the physiological condition, the vasculature exhibits a low level of RUNX2 expression. However, when subjected to bone morphogenetic protein (BMP-2) stimulation, high phosphorus levels, oxidative stress or inflammation, VSMCs undergo osteogenic differentiation. This process is characterized by a reduction in the expression of the specific marker smooth muscle actin (Smooth Muscle Actin Alpha 2, *α*-SMA) and an elevation in RUNX2 expression (71–74). In addition, its downstream osteogenic marker ALP activity is also increased, further contributing to calcification (75).

We successfully induced osteogenesis by treating VSMCs with osteogenic medium, resulting in increased RUNX2 expression, decreased *α*-SMA expression, decreased ALP activity and increased calcium deposition after 7 days. However, Co-incubation with PCSK9i improved these osteogenic indicators, confirming the protective effect of PCSK9i on VSMC osteogenesis. To confirm if PCSK9i’s impact is due to PCSK9, we tested the connection between PCSK9 and osteogenesis in VSMCs. By exposing VSMCs to external PCSK9, we observed that it prompted the cells to adopt an osteogenic phenotype, worsening their calcium salt buildup. This demonstrated that PCSK9 can cause osteogenesis in VSMCs and PCSK9 inhibition is a way to improve this condition.

## 5 Conclusion

Overall, the findings from our MR analysis provide evidence that PCSK9i have the potential to enhance AS, as substantiated by our clinical trials and cellular analyses. Our observations of clinical research indicated that PCSK9i effectively ameliorate CRP levels and mitigate PWV in individuals with ACS, thereby suggesting their capacity to attenuate vascular inflammation and diminish AS. Besides, our cell experiments showed that PCSK9i can ameliorate osteogenesis of VSMCs. Consequently, the significance of incorporating PCSK9i into the therapeutic regimen for ACS patients warrants further emphasis.

## Data availability statement

The datasets presented in this study can be found in online repositories. The names of the repository/repositories and accession number(s) can be found in the article/Supplementary material.

## Ethics statement

The studies involving humans were approved by the ethics committee of the Shanghai East Hospital. The studies were conducted in accordance with the local legislation and institutional requirements. The participants provided their written informed consent to participate in this study.

## Author contributions

LXu: Conceptualization, Formal analysis, Methodology, Writing – original draft, Software. LW: Data curation, Methodology, Writing – review & editing, Software, Validation. YuW: Data curation, Methodology, Writing – review & editing, Validation. YiW: Methodology, Software, Validation, Writing – review & editing. YJ: Data curation, Methodology, Software, Writing – review & editing. PD: Methodology, Supervision, Writing – review & editing. JC: Formal analysis, Methodology, Writing – review & editing. CZ: Methodology, Writing – review & editing. RW: Methodology, Writing – review & editing. TJ: Methodology, Validation, Writing – review & editing. LXi: Formal analysis, Writing – review & editing. JM: Writing – review & editing. JL: Funding acquisition, Resources, Supervision, Writing – review & editing.

## Footnotes

^1^ http://www.phenoscanner.medschl.cam.ac.uk/
