# Supplementary material for: PCSK9 inhibitors ameliorate arterial stiffness in ACS patients: evidences from Mendelian randomization, a retrospective study and basic experiments
Source: Front Med (Lausanne). 2024 May 27;11:1408760. doi: 10.3389/fmed.2024.1408760 (PMC11163136; doi:10.3389/fmed.2024.1408760)
Supplement: Supplementary file 1 [file Data_Sheet_1.docx]

Supplementary Material

# Supplementary Figures and Tables

## Supplementary Figures


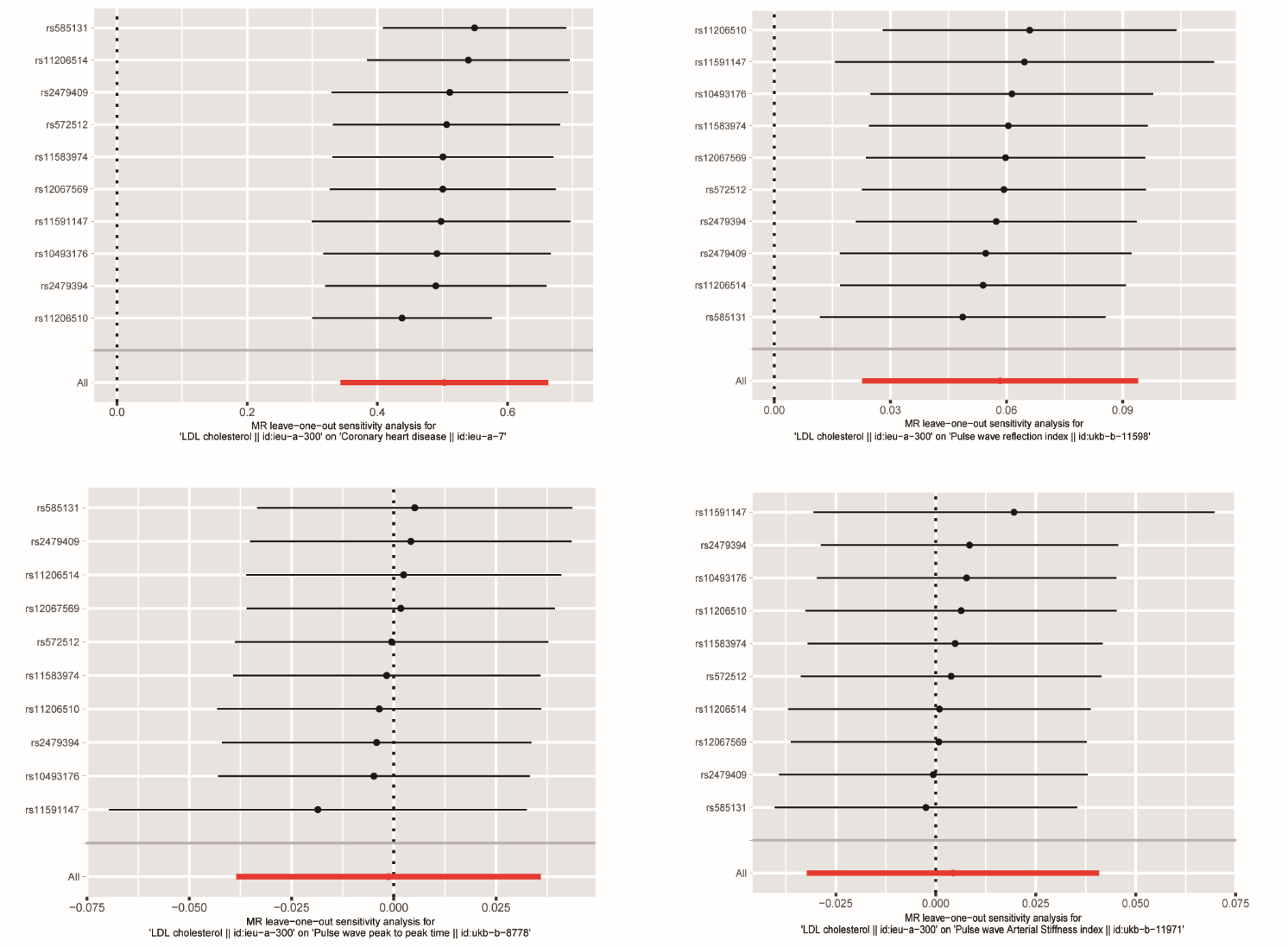


**Supplementary Figure 1.** Results of the leave-one-out method.

## Supplementary tables

Table S1:Information on the datasets used for analyses

Table S2: Harmonized dataset of two-sample Mendelian randomization for the effect PCSK9i on arterial stiffness

Table S3:Heterogeneity and horizontal pleiotropy analyses
